# First Clade Ib Monkeypox Virus Infection Reported in the Americas — California, November 2024

**DOI:** 10.15585/mmwr.mm7404a1

**Published:** 2025-02-13

**Authors:** Vivian Levy, Anna Branzuela, Kristina Hsieh, Shiffen Getabecha, Ricardo Berumen, Kayla Saadeh, Robert E. Snyder, Gillian Marek, Daniel Dodson, Alyssa Newman, Jill K. Hacker, Chantha Kath, Faisal S. Minhaj, Crystal M. Gigante, Shannon Gearhart, Alexander Kallen, Christina L. Hutson, Kathleen Jacobson, Clade I Mpox Response Team, Clade I Mpox Response Team, Robert Avina, Josh Bunao, Jahara Cayabyab, Edwin Chojolan, Erin Epson, Alex Espinosa, Bianca Gonzaga, Monica Haw, Kelly A. Johnson, Awa Keinde, Deidra Lemoine, Kelsey Maccuish, Alexia McGonagle, Rilene Ng, Rachel Piper, Will Probert, Maria Salas, Brandon Stavig, Juliet Stoltey, Orlanda Tafolla, Eric C. Tang, Debra A. Wadford, Jessica Watson, Vivian Wong, Ermias Belay, Shama Cash-Goldwasser, Whitni Davidson, Jiusheng Deng, Marie De Perio, Alida Gertz, David W. Hunter, Aaron Kofman, Ryan Lash, Andrea M. McCollum, A.D. McNaghten, Agam K. Rao, Panayampalli S. Satheshkumar, Emily Sims, Ian Williams, Italo Zecca

**Affiliations:** ^1^San Mateo County Health, San Mateo, California; ^2^Division of Infectious Diseases and Geographic Medicine, Stanford University, Stanford, California; ^3^California Department of Public Health; ^4^Division of High Consequence Pathogens and Pathology, National Center for Emerging and Zoonotic Infectious Diseases, CDC; ^5^Division of Global Migration Health, National Center for Emerging and Zoonotic Infectious Diseases, CDC; ^6^Division of Healthcare Quality and Promotion, National Center for Emerging and Zoonotic Infectious Diseases, CDC.; California Department of Public Health; California Department of Public Health; California Department of Public Health; California Department of Public Health; California Department of Public Health; California Department of Public Health; California Department of Public Health; California Department of Public Health; California Department of Public Health; California Department of Public Health; California Department of Public Health; California Department of Public Health; California Department of Public Health; California Department of Public Health; California Department of Public Health; California Department of Public Health; California Department of Public Health; California Department of Public Health; California Department of Public Health; California Department of Public Health; California Department of Public Health; California Department of Public Health; California Department of Public Health; California Department of Public Health; CDC; CDC; CDC; CDC; CDC; CDC; CDC; CDC; CDC; CDC; CDC; CDC; CDC; CDC; CDC; CDC.

**Summary**

**What is already known about this topic?**

A clade I monkeypox virus (MPXV) outbreak is ongoing in the Democratic Republic of the Congo. Travel-associated clade I MPXV infections have been reported in non-African countries.

**What is added by this report?**

The first reported clade Ib MPXV infection in the Americas was identified via electronic laboratory reporting in California in a U.S. traveler who returned from East Africa. Rapid identification allowed for thorough contact tracing; no secondary cases were identified.

**What are the implications for public health practice?**

Public health authorities should be notified immediately of suspected clade I MPXV infections (e.g., compatible symptoms and travel history, or compatible laboratory results [e.g., presence of nonvariola orthopoxvirus with no detection of clade II MPXV]) to trigger additional testing and enable rapid implementation of transmission-based precautions and other public health interventions.

## Abstract

A clade I monkeypox virus (MPXV) outbreak is ongoing in the Democratic Republic of the Congo; travel-associated clade I MPXV infections have been reported in non-African countries. In November 2024, San Mateo County Health in California identified an electronic laboratory report of polymerase chain reaction results suggestive of clade I MPXV infection in a male traveler who had recently returned from East Africa. After conferring with the California Department of Public Health (CDPH), a county health department worker visited the patient that same day at his home and obtained skin pustule swab specimens for expedited clade I MPXV testing. Clade I MPXV was confirmed the following day by the CDPH Viral and Rickettsial Disease Laboratory. This was the first reported clade I MPXV infection in the Americas. Among 83 identified contacts, five received JYNNEOS vaccine as postexposure prophylaxis. All contacts were monitored for 21 days; no secondary cases were identified. Patients with mpox-compatible lesions or clinical features should receive MPXV testing, and health care providers should immediately notify public health authorities of suspected clade I MPXV infections (e.g., mpox manifestations and travel history to an area with ongoing clade I MPXV transmission) or upon receiving a nonvariola orthopoxvirus DNA detected, clade II MPXV DNA undetectable test result to trigger additional testing and facilitate the rapid implementation of transmission-based precautions and other preventive public health interventions.

## Introduction

A clade I monkeypox virus (MPXV) outbreak in the Democratic Republic of the Congo (DRC) has been ongoing since 2023 amidst a worldwide outbreak of clade II MPXV that began in 2022. A newly emerged clade I subclade (clade Ib MPXV) has been spreading through close person-to-person contact in sub-Saharan African countries where MPXV is not endemic (*1*,*2*). Clade I MPXV infections have historically been associated with a higher case fatality rate (1.4%–11%) than have clade II MPXV infections (0.1%–3.6%) (*3*,*4*); however, recent estimates for clade Ia MPXV in patients who have received optimal supportive care were lower (1.4%–1.7%) (*5*), as were estimates for clade Ib MPXV in DRC and other east African countries where only clade Ib has been identified (<1%) (*3*,*6*). Isolated travel-associated clade Ib MPXV infections have also been reported outside of Africa; none have resulted in serious illness or death (*7*). In November 2024, a clade Ib MPXV case was identified in an otherwise healthy male patient in California. This report describes the clinical features of and public health response to the first identified clade Ib MPXV infection in the Americas.

## Methods

In November 2024, San Mateo (California) County (SMC) Health identified an electronic laboratory report (ELR) from a commercial laboratory with polymerase chain reaction (PCR) results suggestive of clade I MPXV infection (i.e., nonvariola orthopoxvirus [NVO] DNA detected, clade II MPXV DNA undetectable) via California’s statewide ELR processing and case management systems, the Surveillance and Public Health Information Reporting and Exchange (SaPHIRE), and the California Reportable Disease Information Exchange. SMC Health alerted the California Department of Public Health (CDPH) Viral and Rickettsial Disease Laboratory (VRDL), that obtained PCR cycle threshold (Ct) results from the commercial laboratory, which supported the possible diagnosis of clade I MPXV. The same day, SMC Health visited and interviewed the patient to assess mpox risk factors, including travel history and clinical signs and symptoms; to collect additional lesion swabs for confirmatory clade I MPXV testing; and to guide any requisite public health activities in response to these findings. Clade I MPXV was confirmed by real-time PCR assays developed at VRDL, including a diagnostic triplex MPXV assay based on panMPXV, clade Ia MPXV, and clade II MPXV gene targets (*8*), and a newly developed surveillance assay (panMPXV, clade I, clade II, and panOrthopoxvirus targets) as well as clade Ib–specific PCR assay at CDC, and whole genome sequencing from both VRDL and CDC. This activity was reviewed by CDC, deemed not research, and was conducted consistent with applicable federal law and CDC policy.* Written permission from the patient was obtained to share details of this case.

## Investigation and Results

### Case Investigation

**FIGURE 1:**
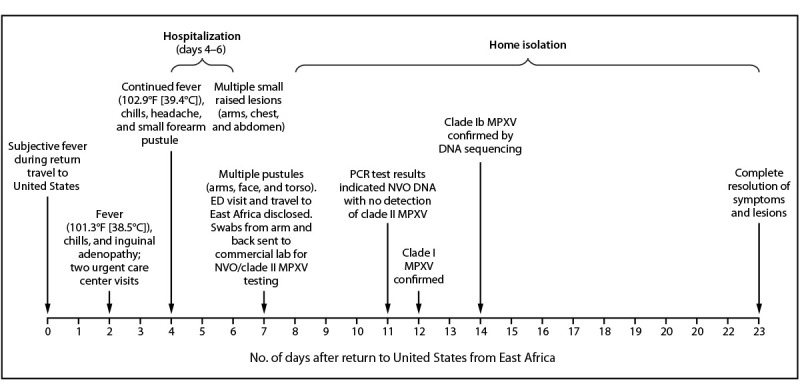
Signs and symptoms; clinical evaluation, findings, and management*; and laboratory results in a case of clade Ib monkeypox virus infection in a traveler to East Africa^†^ — California, 2024 **Abbreviations:** ED = emergency department; MPXV = monkeypox virus; NVO = nonvariola orthopoxvirus; PCR = polymerase chain reaction. * Patient received oral antibiotics on two separate occasions, beginning on days 2–3, and again after hospital discharge on days 6–11. Intravenous antibiotics were administered during the patient’s hospitalization. **^†^** The patient’s travel to East Africa was ascertained at the time of the ED evaluation on day 7. Swabs were sent for testing at that time.

Interviews with the traveler by medical providers and SMC Health revealed that before departure for Africa from the United States, he reported the presence of a small “ingrown hair” in the pubic area, which became more tender and swollen over the course of his 1-week trip in East Africa. He experienced subjective fever during his return to the United States via commercial airline (day 0) (Figure 1). On day 2 after returning to the United States, he was evaluated at two urgent care facilities for progressive signs and symptoms, including fever, shortness of breath, fatigue, and nausea, as well as new unilateral inguinal adenopathy. On exam, a 1 cm purulent, draining ulcer at the base of the penis was noted, with multiple shotty, tender left inguinal lymph nodes; no other rash or lesions were present. His travel history was not elicited during either of these urgent care evaluations. Results of diagnostic tests for other infectious diseases were negative, and he was prescribed oral trimethoprim-sulfamethoxazole and amoxicillin-clavulanic acid twice daily for presumed pubic folliculitis.

Two days later (day 4), he was evaluated at an emergency department (ED) with persistent fever, chills, and headache. The pubic ulcer appeared to be healing, but lymphadenopathy was still present, as was a new small pustular lesion on his right forearm. Laboratory tests showed elevated erythrocyte sedimentation rate and elevated c-reactive protein that indicated inflammation and acutely elevated creatinine; he was hospitalized for presumed inguinal cellulitis that had not responded to oral antibiotics and acute kidney injury.

During administration of intravenous (IV) vancomycin in the ER, he experienced flushing and a nonpruritic rash on his right arm, symptoms that resolved when the infusion rate was decreased. IV broad-spectrum antibiotics were also empirically administered. Over the next few days, a pruritic rash described as “tiny bumps” appeared on his arms, chest, and abdomen; the rash was initially thought to be medication-related and reportedly improved following topical application of 2.5% hydrocortisone cream. On day 6, he was clinically improved, his fever had resolved, and his kidney function was normal. He was discharged on oral doxycycline and cefpodoxime.

**FIGURE 2:**
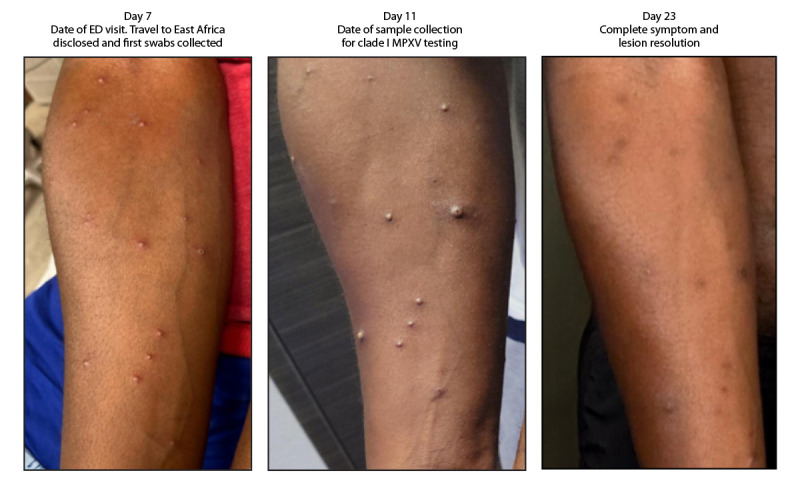
Progression of lesions in a patient with clade Ib monkeypox virus infection 7, 11, and 23 days after return to the United States from East Africa — California, 2024 Photos/Anna Branzuela (photos reproduced with the patient’s permission) **Abbreviations**: ED = emergency department; MPXV = monkeypox virus.

The following day, the inguinal pain worsened, and discrete papular, vesicular, and pustular lesions appeared on his face, back, arms, and legs (Figure 2), prompting another ED visit. It was at this visit, 7 days after his return to the United States, that his travel to East Africa was first elicited. A mild leukocytosis was noted, and a computerized tomography scan demonstrated continued inguinal lymphadenopathy but excluded loculated fluid collections. The ulcer at the base of the penis was dry with mild surrounding induration and no drainage. Pustules on the patient’s arm and back were swabbed and sent for varicella zoster virus (VZV) and MPXV testing. 

On day 11, the commercial laboratory reported detection of NVO DNA (Ct = 25 [reactive]) and an absence of clade II MPXV DNA, suggesting possible clade I MPXV. VZV results were negative. Lesions from the patient’s arm and leg were reswabbed on day 11 by SMC Health, and testing at CDPH VRDL the following morning revealed clade I MPXV DNA (diagnostic panMPXV positive but clade Ia and clade II negative; surveillance panMPXV/clade I MPXV/panOrthopoxvirus positive but clade II MPXV negative). On day 14, whole genome sequencing from the day 11-specimens conducted at CDPH VRDL and CDC confirmed clade Ib MPXV.^†^

The patient reported having attended social gatherings and received a full-body massage while traveling in East Africa, and he reported no sexual contact during travel. The patient isolated at home until all lesions resolved as verified by the local health department physician’s clinical exam at the patient’s home on day 23.

### Identification of Contacts

A total of 83 contacts were identified and stratified into the following risk groups^§^ (*9*): one household contact (high-risk), four travel companions (uncertain or minimal risk), 10 flight contacts who were seated ≤6 feet of from the patient (uncertain or minimal risk), and 68 health care personnel (HCP) (three intermediate, 56 uncertain or minimal, and nine no identifiable risk). Most contacts (77) were enrolled in twice-daily automated symptom monitoring for 21 days; six flight contacts were unable to be reached. Six HCP reported incidental lesions on their necks, backs, or legs; these lesions were not characteristic of mpox, and no orthopoxvirus DNA was detected in swabs collected from these lesions. The household contact and four travel companions received JYNNEOS postexposure prophylaxis. No secondary cases were identified.

### Public Health Response

Before detection of this first clade I (i.e., clade Ib) MPXV infection in the Americas, CDC and public health partners had begun to adapt clade II MPXV surveillance efforts to support clade I MPXV preparedness. These included leveraging of commercial and public health laboratory testing as surveillance for clade I (i.e. NVO positive, clade II negative), implementation of additional laboratory-developed tests within public health laboratories and CDC (including the panMPXV and clade I MPXV PCR assays at CDPH VRDL), and the development and dissemination of public health and clinical guidance via CDC Health Action Network (HAN) and California Health Action Network (CAHAN) publications and dissemination of information to the laboratory community through CDC Laboratory Outreach Communication Systems (LOCS) messages. In addition, when the clade I MPXV outbreak in Africa was first recognized and reported internationally, CDPH created and implemented a statewide surveillance system to monitor ELR data daily for NVO/clade II MPXV PCR results that could be indicative of a clade I MPXV infection (i.e., nonvariola orthopoxvirus DNA detected, clade II MPXV DNA undetectable). After identification of this clade Ib MPXV infection, public health interventions were quickly implemented, including continuing the ongoing daily monitoring for additional suspicious ELRs during contact tracing, clear and consistent communication and guidance through HAN and CAHAN reports, and ongoing additional communications with local health departments and clinicians since this first detection (*6*).

## Discussion

This case represents the first clade I MPXV (specifically, clade Ib) infection detected in the Americas. Consistent with other travel-associated clade Ib MPXV infections outside Africa, the infection did not result in severe illness, and the patient recovered without sequelae (*7*). In late November 2024, the Public Health Agency of Canada confirmed the first case of clade I MPXV in Manitoba (*10*). In January 2025, another travel-associated clade I mpox case in the United States was reported in Georgia; that patient’s illness was also not severe, with no reported subsequent cases among contacts. In February 2025, two more travel-associated clade Ib mpox cases were reported, one each in New Hampshire and New York State; both patients are isolating and recovering at home. CDC and local public health partners continue to conduct clinical, epidemiological, laboratory, and wastewater surveillance that will enable rapid identification of, and response to, MPXV in the United States.

### Limitations

The findings in this report are subject to at least one limitation. Although it is assumed that the patient’s infection was acquired in East Africa, and the “ingrown hair,” present before the patient’s departure from the United States was not characteristic of mpox, it was not tested for MPXV DNA. That lesion was the only reported symptom before his U.S. departure, and the remainder of the clinical course and timeline were consistent with exposure to and acquisition of MPXV infection in East Africa. Thus, although the break in skin might have served as a portal of entry, increasing the risk for MPXV infection while traveling, it is unlikely that this patient was infected with MPXV before travel to East Africa. This finding is also consistent with there being no other MPXV infections in SMC or in nearby counties within 21 days of onset of this patient’s ulcer that were not already confirmed to have been attributed to clade IIb MPXV.

### Implications for Public Health Practice

Given the possibility of future travel-associated clade I MPXV cases, public health partners should consider implementing electronic alert systems similar to that in California that scan ELR data for NVO-positive/clade II MPXV–negative results to identify suspected clade I MPXV infections. HCP should also promptly obtain a thorough travel history from all patients with an acute infection and consider mpox in persons with lesions or other clinical signs and symptoms compatible with mpox.^¶^ HCP who see patients with clinical signs of mpox and who meet at least one of the epidemiologic criteria for clade I MPXV infection** should immediately notify public health authorities to facilitate testing and the implementation of treatment and prevention interventions.

## Footnotes

* 45 C.F.R. part 46.102(l)(2), 21 C.F.R. part 56; 42 U.S.C. Sect. 241(d); 5 U.S.C. Sect. 552a; 44 U.S.C. Sect. 3501 et seq.

† https://www.ncbi.nlm.nih.gov/nuccore/PQ662929.1/

§ https://www.cdc.gov/mpox/php/monitoring/index.html

¶
https://www.cdc.gov/mpox/hcp/clinical-signs/index.html

**
https://www.cdc.gov/mpox/hcp/case-definitions/index.html#cdc_generic_section_5-epidemiologic-criteria
